# Genome-Wide Profiling Reveals That Herbal Medicine Jinfukang-Induced Polyadenylation Alteration Is Involved in Anti-Lung Cancer Activity

**DOI:** 10.1155/2017/5326909

**Published:** 2017-11-01

**Authors:** Yao Kou, Guoqing Li, Jinhui Shao, Cong Liu, Jun Wu, Jun Lu, Xiaodong Zhao, Jing Tian

**Affiliations:** ^1^Key Laboratory of Resource Biology and Biotechnology in Western China, Ministry of Education, College of Life Sciences, Northwest University, 229 Taibai Road, Xi'an 710069, China; ^2^Shanghai Center for Systems Biomedicine, School of Biomedical Engineering, State Key Laboratory on Oncogene and Bio-ID Center, Shanghai Jiao Tong University, 800 Dongchuan Road, Shanghai 200240, China

## Abstract

Alternative polyadenylation (APA) plays an important role in regulation of genes expression and is involved in many biological processes. As eukaryotic cells receive a variety of external signals, genes produce diverse transcriptional isoforms and exhibit different translation efficiency. The traditional Chinese medicine (TCM) Jinfukang (JFK) has been effectively used for lung cancer treatment. In this study, we investigated whether JFK exerts its antitumor effect by modulating APA patterns in lung cancer cells. We performed a genome-wide APA site profiling analysis in JFK treated lung cancer cells A549 with 3T-seq approach that we reported previously. Comparing with those in untreated A549, in JFK treated A549 we observed APA-mediated 3′ UTRs alterations in 310 genes including 77 genes with shortened 3′ UTRs. In particular, we identified* TMEM123*, a gene involved in oncotic cell death, which produced transcripts with shortened 3′ UTR and thus was upregulated upon JFK treatment. Taken together, our studies suggest that APA might be one of the antitumor mechanisms of JFK and provide a new insight for the understanding of TCM against cancer.

## 1. Introduction

Alternative polyadenylation (APA) has been emerged as an important mechanism for posttranscriptional regulation of genes in eukaryotes [1]. Posttranscriptional regulation of gene expression is controlled by 5′-end capping, 3′-end poly (A) tail adding, and a series of alternative splicing processing, in order to produce the functional mature mRNAs. The 3′-end processing involves the cleavage of transcript and the addition of poly (A) tail at poly (A) site (PAS) [2]. A gene in different tissues and at different stages of the cells produces distal or proximal PAS in 3′ UTRs by APA which can generate different isoforms based on the variable splicing sites [3]. 3′ UTRs of mRNA, though not encode proteins, have important regulatory roles in genes expression. Previous studies showed that transcript isoforms with short 3′ UTRs generated by APA exhibited increased stability of mRNA and produced 10 times more protein due to loss of miRNA binding sites in 3′ UTRs, which made mRNA escape the negative regulation of miRNA [4]. In addition to regulating gene expression, APA modulation which is tissue and stage specific is also associated with various biological processes, such as proliferation, development, cellular differentiation, and neuron activation [4–6]. Recent studies have showed that APA events were also very important for the transformation and proliferation of cancer cells. Lin et al. performed a comprehensive mapping over 1 million APA sites and found that signaling cascades mediated by cell–cell and cell–extracellular matrix contact are potentially key targets of APA in different cancers, including lung, liver, kidney, breast, and colon cancers [7].

Nonsmall cell lung cancer (NSCLC) is the most common type of lung cancer which becomes the leading cause of cancer-related death worldwide. The 5-year relative survival rate for people with lung cancer is only 18% [8]. Treatments of NSCLC are based mainly on the stage of cancer, including surgery, chemotherapy, and radiotherapy, which have been applied in clinical interventions to help patients live longer by relieving symptoms [9, 10]. However, the patients' survival time and quality of life have not been improved significantly.

Traditional Chinese Medicine (TCM) Jinfukang (JFK) has been clinically employed in the lung cancer treatment for more than ten years [11]. Recent studies suggested that JFK could increase survival rate and quality of life of NSCLC patients, due to improving the patients' own immune systems when combined with chemotherapy [12, 13]. Studies in animal model also showed that JFK could inhibit the development of lung cancer by 49.2% versus control in mice [14].

Although the previous studies suggested that JFK antitumor mechanisms may include cell cycle [15], apoptosis [15], and epigenetic regulation [16], there are still many unknowns in this area. Here we performed the 3T-seq technology which was established in our lab [17] to analyze the 3′ UTRs profiling of genes regulated by APA in JFK treated NSCLC cancer cells and examined the JFK antitumor activities at the posttranscriptional regulation levels.

## 2. Materials and Methods

### 2.1. JFK Extraction

The mixture of herbal medicine JFK [14] was refluxed with 70% ethanol at 80°C for 1 h, filtered with 0.22 *μ*m membrane, and stored at 4°C.

### 2.2. Cell Culture

NSCLC cell line A549 was obtained from the Shanghai Institute of Biochemistry and Cell Biology. A549 cells were cultured in RPMI-1640 medium (Gibco, USA) containing 10% fetal bovine serum (GIBCO, USA), incubated at 37°C with a humidified atmosphere of 5% CO2. The cells were subcultured when they reached approximately 90% confluence.

### 2.3. Cell Viability Analysis

A549 cells were cultured in 96-well plates at a density of 5000 cells/well overnight and incubated with different doses of JFK (30 *μ*g/ml, 45 *μ*g/ml, 60 *μ*g/ml, 90 *μ*g/ml, 120 *μ*g/ml, 150 *μ*g/ml, and 200 *μ*g/ml) for another 24 h. Cell viability determined by Cell Counting Kit 8 (Dojindo, Japan). Cell viability was calculated by measuring the optical absorbance at 450 nm with a microplate reader (Bio-Tek, USA). Absorbance of the control sample was set at 100% viability, and each experiment was repeated at least three times.

### 2.4. PI Staining

A549 cells were fixed, stained with Propidium Iodide (PI) (Sigma, USA) after 24 h of culturing, and observed by fluorescence microscopy (Nikon, Japan) at the excitation wavelength of 536 nm. Cells were considered as being dead if the staining of cells was red and as being not dead if the staining of cells was slight red or there was no staining.

### 2.5. Cell Death Analysis

Determination of phosphatidylserine (PS) and membrane integrity was performed by Annexin V-FITC/PI Apoptosis kit (Zoman, China). A549 cells were harvested by trypsin digestion and washed with PBS, stained with Annexin VFITC/PI, and analyzed by flow cytometry (BD LSRFortessa, USA).

### 2.6. Library Preparation with 3T-Seq

Total RNA was extracted with TRIzol Reagent (Ambion, USA) according to the manufacturer's instructions and dissolved in the RNase free water. The RNA was treated with TURBO DNA-free™ Kit (Life technologies, USA) to remove genomic contamination. 3T-seq assay was performed as described previously [17]. In brief, the reverse transcription was carried by using superscript III (Invitrogen) in M280 Streptavidin beads (Invitrogen) which were bound with biotin modified GsuI-Oligo (dT) 18 primers. dCTP was replaced by 5-methylated-dCTP during second strand synthesis. The resulting dsDNA was fragmented to ~200–500 bp with the time dependent enzyme Fragmentase (Thermo). The 3′ terminal fragments were released from magnetic beads by restriction endonuclease GsuI (Thermo) digestion, ligated with Illumina P5/P7 sequencing adapters and subjected to high throughput sequencing performed with the Illumina Hiseq 2500.

### 2.7. Bioinformatics Analysis

Data processing method was similar to the previous report [14]. The original data was screened and trimmed with C++ scripts and the processed reads were mapped to the human genome (hg19) with bowtie2 (version 2.1.0 by Ben Langmead) [18]. The mapping results were iteratively clustered in order to construct the poly (A) sites [19]. The 3′ UTRs switching for each gene among the two samples was detected by measuring the linear trend alternative to independence and *P* value less than 0.05 was regarded as significant. The false discovery rate was estimated with R software. Functional analysis of the genes with altered 3′ UTRs was performed by using the online software PANTHER [20].

### 2.8. Quantitative Real-Time PCR Validation

RNA was isolated from JFK treated and untreated A549 cells using TRIzol Reagent (Ambion, USA) as described before. 1 *μ*g of RNA was reverse transcribed using the RevertAid First Strand cDNA Synthesis Kit (Thermo) and random hexamers. Quantitative real-time PCR (qRT-PCR) was performed with SYBR Green PCR Master Mix (Kapa Biosystems, USA). Reactions were run in triplicate and normalized against *β-actin*. Primer sequences were as follows:

 
*β*-actin forward primer, 5′-AGTTTGTGGCCCTGATCTGT-3′ and reverse primer, 5′-CATTCTTGTCCACACGTCCA-3′;
 
*TMEM123*, forward primer, 5′-TGCAAGTGCTTCTCCAAACTC-3′ and reverse primer, 5′-GCCTTTGGTGGGATGAGTAG-3′;
  3′ UTR of* TMEM123* mRNA, forward primer, 5′-CATATAATGTACAGTGTATTACG-3′; and reverse primer, 5′-ACCATTGTATGAACGGTCTAT-3′.

### 2.9. Statistics Analysis

Each experiment was repeated at least three times, and data were expressed as means ± SD. Differences among triplicate were analyzed by Student's *t*-test. Significant differences were considered to be those with *P* < 0.05.

## 3. Results

### 3.1. Morphology and Viability Changes on JFK Treated A549 Cells

**Figure 1:**
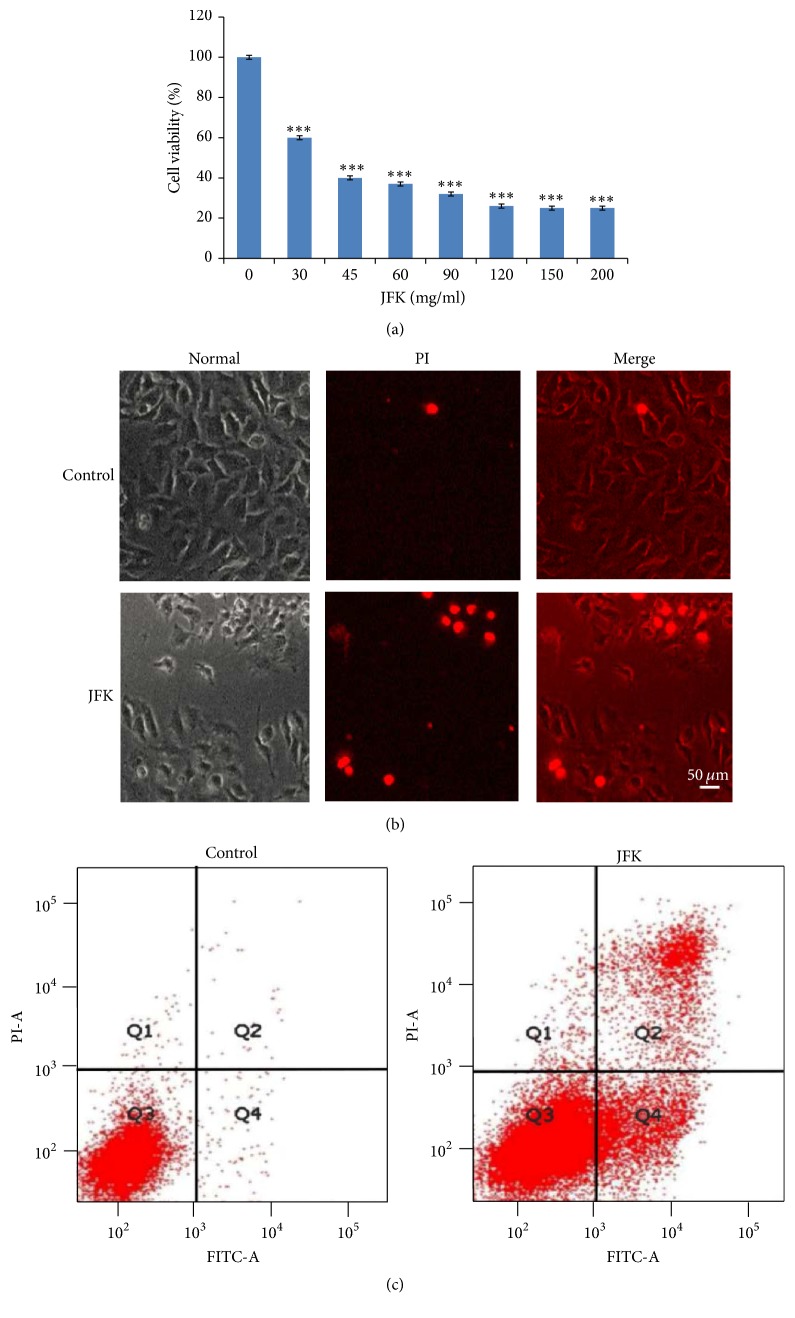
Treatment with JFK induced cell death in A549 cells. (a) Inhibitory effect of JFK on viability of A549 cells. A549 cells were treated with JFK (30 *μ*g/ml, 45 *μ*g/ml, 60 *μ*g/ml, 90 *μ*g/ml, 120 *μ*g/ml, 150 *μ*g/ml, and 200 *μ*g/ml, resp.) for 24 h; the viable cells were determined by CCK8 assay. (b) Observation of A549 cells under optical microscope and fluorescence microscope stained with PI for detecting dead cells. Scale bar: 50 *μ*m. (c) A549 cells were treated with JFK, and the apoptotic progression was determined by flow cytometry. Values in (a) represent means ± SE of data from three independent experiments, ^*∗∗∗*^*P* < 0.001.

Cell viability was decreased significantly after 24 h incubation with 30 *μ*g/ml JFK and shown in a dose-dependent manner (Figure 1(a)). We chose 30 *μ*g/ml JFK which caused obvious cell death after 24 h incubation with JFK for all future experiments. Cell morphology was also changed from the oval or spindle type to variable radial cells after 24 h incubation with JFK (Figure 1(b)). To further understand the effects of JFK on A549 cells, PI staining was performed by flow cytometry analysis. As shown in Figure 1(c), dead cells were colored red when stained with PI; comparing to the untreated groups, the number of dead cells was increased after JFK treatment, suggesting that JFK could induce cell death in A549 cells.

### 3.2. APA Loci Capture in the Whole Genome Range Based on 3T-Seq

**Figure 2:**
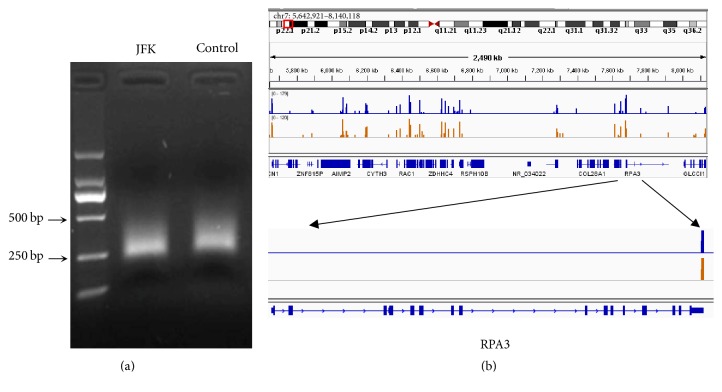
Identification of APA sites in A549 cells treated with JFK. (a) Gel electrophoresis of the 3T-seq library; (b) a genomic view of APA sites defined by 3Tseq in IGV genome browser. Blue track: JFK; Yellow track: Control.

Library was checked by electrophoresis (Figure 2(a)), with the bands mainly distributed around the region of 200–500 bp. Raw data from high throughput sequencing was analyzed by Integrative Genomics Viewer (IGV) software (Figure 2(b)). 5,945,544 and 7,207,234 reads were generated with TT/AA tag from JFK treated and untreated A549 cells, in which 2,625,908 and 3,140,671 reads had unique location in genome, respectively.

**Figure 3:**
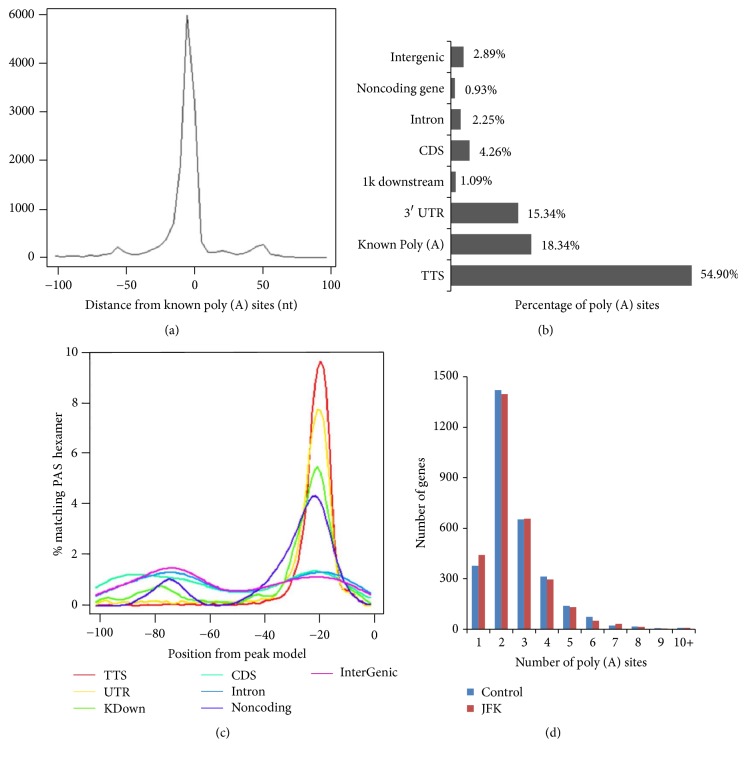
Characterization and comparative analysis of APA sites in A549 cells treated with JFK. (a) Distribution of 3T-seq reads across the gene body. (b) Genomic locations of poly (A) sites. (c) Position-specific distributions of PAS signal hexamer for poly (A) sites. (d) Statistics of genes with various number of detected poly (A) sites.

For the identification of APA sites, we took a “snowball” method to cluster reads. Reads within a distance of 25 nt belong to one cluster [21]. Totally we identified 51,090 PAS sites from 25,817 and 25,273 poly (A) sites in JFK treated and untreated A549 cells, respectively. Most of the APA loci were obtained at the annotated transcription termination sites (TTS), as shown in Figure 3(a). The screening of the results was shown in Table 1.

Among 51,090 identified poly (A) sites, 54.9% of PAS were mapped to the known UCSC TTS sites, 18.34% mapped to poly (A) database, and 15.4% mapped to the known 3′ UTR region which may be the novel PAS, as shown in Figure 3(b). It is believed that PAS formation mechanism is related to a hexamer motif AAUAAA/AUUAAA, which is located at the PAS upstream 20–30 nt in 3′ UTR [17]. PAS hexamer could be found in all APA sites, proving that the definition of the PAS was efficient (Figure 3(c)). Numbers of the genes with alternative PAS were shown in Figure 3(d), and genes with two PAS were more than others in A549 cell.

### 3.3. 3′ UTRs Analysis between Gene Clusters in JFK Treated and Untreated Cells

**Figure 4:**
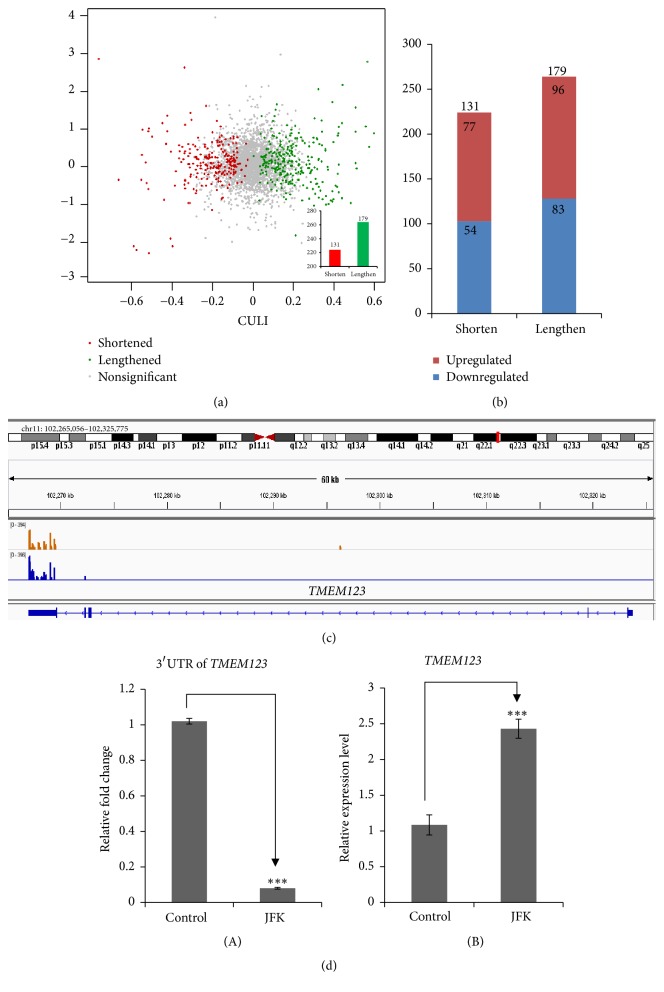
APA sites variations in A549 cells treated with JFK compared to control. (a) Scatter diagram of genes with differential APA defined by CULI. (b) Composition of downregulated and upregulated genes in A549 cells treated with JFK. (c)* TMEM123* transcript isoforms with alternative poly (A) sites in JFK treated (Blue) and untreated (Brown) A549 cells. (d)(A) Shortened 3′ UTR of TMEM123 mRNA was verified by qRT-PCR. (B) Verification of TMEM123 expression level by qRT-PCR. Values in (d) represent means ± SE of data from three independent experiments, ^*∗∗∗*^*P* < 0.001.

Linear trend method was used [21] for homogenization treatment of genes containing multiple PAS. The cancer 3′ UTR length index (CULI) was used for quantitative indication of 3′ UTR length of a given gene. If CULI is positive, 3′ UTR is lengthened in JFK treated cells compared with the untreated group. On the contrary, a negative CULI indicated shortened 3′ UTR. 310 genes were significantly changed in the length of 3′ UTRs. Among 310 genes, CULI as negative were 131 (42%) and CULI as positive were 179 (58%). The overall change trend is shown in Figure 4(a). The number of genes with different PAS represents the diversity of APA. The distribution of PAS was shown in Figure 4(b).

### 3.4. Functional Analysis of Differentially Expressed Genes

When the 3′ UTRs are altered by APA, miRNA binding sites in the 3′ UTRs will be changed accordingly. When 3′ UTRs becomes shorter, some miRNA binding sites may be cut off; expression levels of genes can be increased due to escaping from miRNA silencing. According to the comparative analysis of APA position, 77 genes were selected according to their upregulated expression and shortened 3′UTR of mRNA after JFK treatment.

Gene ontology analysis of the 77 affected genes revealed that the 3′ UTR of* TMEM123*, a gene mediating cell death through oncosis, was significantly shortened. Analysis of* TMEM123* in IGV was shown in Figure 4(c)*. TMEM123* encodes a type I transmembrane protein of 189 amino acids, with a high content of threonine and serine residues in the extracellular domain. TMEM123 protein is a prooncosis receptor which induces membrane injury. When exposed to anti-PORIMIN antibody, TMEM123 protein can be crosslinked on the cell surface and induce oncosis. Expression of TMEM123 also causes loss of cell adhesion, cell membrane injury, and cell death [22].

### 3.5. 3′ UTR Shortening of* TMEM123* mRNA

We designed primers which were located within the shortened region in* TMEM123* mRNA 3′ UTR after JFK treatment. As showed in Figure 4(d)(A), with JFK treatment,* TMEM123* exhibited 3′ UTR shortening (*P* < 0.001).

### 3.6. Verification of* TMEM123* mRNA Level

The examination of* TMEM123* expression level was shown in Figure 4(d)(B). With 3′UTR shortened, the expression level of* TMEM123* was remarkably increased (*P* < 0.001), indicating that the expression of* TMEM123 *was affected by APA regulation.

## 4. Discussions

APA events have emerged as the widespread phenomenon in human genome by generating different transcript isoforms with alternative 3′ end. In cells, APA is involved in the regulation of mRNA stability, translation activity, and intracellular localization of transcription factors by producing 3′ UTRs at different length [4, 7, 12]. Previous report observed that several oncogenes preferentially generate short 3′ UTR in cancer cell, which might abolish miRNA binding sites in 3′ UTRs and escape miRNA regulated programmed cell death [4]. Genome-wide analyses of APA sites have revealed the widespread shortening of 3′ UTRs in both cancer cell lines and cancer tissues [4]. It provides a new insight to better understand tumorigenesis and antitumor mechanisms.

Although the effectiveness of traditional Chinese medicine (TCM) on tumor therapy has been well recognized in clinical practice, the cellular and molecular mechanisms are rarely known, mainly due to the complex composition of TCM. As an effective medicine to treat lung cancer, JFK has been clinically used for more than ten years. Previous studies have already showed that JFK could modulate genes expression involved in the cell cycle, apoptosis, and epigenetic regulation [15]. However, there are few studies on posttranscriptional regulation field. In this study, by performing a 3T-seq technology, we were able to analyze JFK antitumor mechanism on posttranscriptional levels. We identified 77 genes with shortened 3′ UTRs in JFK treated lung cancer A549 cells. Gene ontology analysis of these affected genes revealed* TMEM123* might induce cell death in JFK treated lung cancer A549 cells. Further analysis by qRT-PCR demonstrated that 3′ UTR shortening could enhance gene expression. As mentioned above, oncogenic activation derived from APA is partially due to the escape of miRNA-mediated repression; it is likely that the APA-mediated* TMEM123* 3′ UTR shortening causes cell death and promotes lung metastasis.

Previous studies of JFK demonstrated that JFK induced lung cancer cell death by regulating some apoptotic gene expression, such as AIFM2 [23], IL-2 [24], and Bcl2 [16]. In this study, we proposed that the programmed cell death induced by JFK may also be caused by* TMEM123*-mediated oncosis. But we could not observe swollen cells under microscopy due to the severe cell death. Therefore, we supposed that the JFK induced lung cancer cell death, predominately via apoptosis, and, to a certain extent, might be via oncosis. As the composition of Chinese herbal medicine is very complex, the separation and purification of more effective components and the molecular mechanism study of these single components are worthy of being expected.

3T-seq technology is a robust approach for global APA profiling. With 3T-seq, PAS could be precisely positioned by TT/AA tag at 3′-end of mRNA and a high quality of TT/AA tag library could be established. The development and application of high throughput sequencing technology makes whole genome scope of APA profiling as an effective strategy to study the posttranscriptional regulation. By analyzing gene expression and APA alternation, we indicated the antitumor mechanism of JFK might involve in the posttranscriptional regulation.

With the incidence of lung cancer increasing year by year, the cure rate of lung cancer was still less than 10%. The treatments, including surgery chemotherapy, and radiotherapy, bring great burden to the patient's physical and mental health. With the progress and development of TCM, more people in China choose a combination of traditional Chinese and Western medicine to treat cancer. However, the complexity of TCM makes it difficult to get clear targets of drugs. In recent years, clinical research on the Chinese medicine increased, but the molecular level of research has yet to be further strengthened. Our work provides firstly a feasible research method and a new perspective for the study of the molecular mechanism of TCM from the whole genome-wide range at the posttranscriptional regulation levels. But only alteration of genes expression may not very persuasive. Further study should be involved with the protein expression. Immunological methods and* in vivo* studies may be used to explain the tumor inhibition mechanism with JFK treatment.

## Figures and Tables

**Table 1:** Summary statistics of sequencing data from Illumina Hiseq2500.

	Control	JFK
Raw data	13513778	18691095
TT trim	5945544	7207234
Mapping	5295732	6371247
Unique	2738290	3271990
Mapped to nuclear genome	2625908	3140671
Passed internal priming filter	2310394	2888610
Poly(A) sites	25817	25273

## Abbreviations

APA: Alternative polyadenylation
IGV: Integrative Genomics Viewer
JFK: Jinfukang formula
NSCLC: Nonsmall cell lung cancer
PAS: Alternative polyadenylation site
PI: Propidium iodide
TCM: Traditional Chinese medicine
TTS: Transcription termination sites.
